# Low hepatitis B vaccine uptake among surgical residents in Cameroon

**DOI:** 10.1186/1755-7682-7-11

**Published:** 2014-03-14

**Authors:** Jean Jacques N Noubiap, Jobert Richie N Nansseu, Karen K Kengne, Ambroise Wonkam, Charles S Wiysonge

**Affiliations:** 1 Faculty of Medicine and Biomedical Sciences, University of Yaoundé I, Yaoundé, Cameroon; 2 Internal Medicine Unit, Edea Regional Hospital, PO Box 100, Edea, Cameroon; 3 Mother and Child Centre, Chantal Biya Foundation, Yaoundé, Cameroon; 4 Division of Human Genetics, Faculty of Health Sciences, University of Cape Town, Cape Town, South Africa; 5 Center for Evidence-based Health Care, Department of Interdisciplinary Health Sciences, Faculty of Medicine and Health Sciences, Stellenbosch University, Stellenbosch, Western Cape, South Africa

**Keywords:** Hepatitis B vaccine, Healthcare workers, Cameroon

## Abstract

**Background:**

Hepatitis B virus (HBV) infection is one of the most serious occupational hazards faced by healthcare workers. Surgical personnel are particularly at risk. HBV infection is preventable by vaccination, but no previous study has assessed HBV vaccination coverage among healthcare workers in Cameroon. We assessed knowledge of risk factors of HBV infection, awareness of HBV vaccine, and vaccination status of surgical residents in Cameroon.

**Methods:**

A structured pretested questionnaire was administered to 49 of the 70 surgical residents in Cameroon during the 2011–2012 academic year.

**Results:**

Since the beginning of their residency program, 28 (57.1%) had had at least one accidental exposure to blood, with a median of 2 (range 1 to 25) exposures. Most of them had a good knowledge of risk factors for HBV infection. Although 98.0% (n = 48) were aware of the HBV vaccine, and 89.8% (n = 44) knew that they were at high risk of infection, only 24.5% (n = 12) had received a full course of at least three doses of the vaccine. In addition, only 33.3% (4/12) underwent post-vaccination testing to confirm a good immunological response (and thus effective protection against HBV infection). Among the 53.1% (n = 28) who had never had any dose of HBV vaccine, the main reasons for not being vaccinated were lack of time (38.5%), lack of money to pay for vaccine (23.1%), and lack of sufficient information on the vaccine (19.2%). Only 20.4% (n = 10) had been sensitized by their training institutions about the importance of HBV vaccination.

**Conclusion:**

There is a low HBV vaccine uptake among surgical residents in Cameroon. As part of occupational safety measures, complete HBV vaccination should be strongly recommended and offered to surgical trainees before the beginning of their training program.

## Introduction

Healthcare workers are at occupational risk of exposure to blood-borne pathogens. There are more than 20 known blood-borne pathogens, but those of major public health significance are the human immunodeficiency virus (HIV), hepatitis C virus (HCV), and hepatitis B virus (HBV) [1]. HBV is more infectious than the other blood-borne pathogens, with a 30% risk of contamination after exposure by a single needle stick injury compared to 3% for HCV and 0.3% for HIV [2]. HBV is transmitted by exposure to infectious blood or body fluids such as semen and vaginal fluids, and perinatally from mother to child [3].

It is estimated that more than two billion people worldwide have been infected by HBV at one point of their lives and about 350 million of them suffer from chronic HBV infection; mainly liver cirrhosis and hepatocellular carcinoma [4]. The carrier rate of HBV in Cameroon is estimated at 10% of apparently healthy adults [5]. In the United States of America the incidence of HBV infection among all healthcare workers is estimated to be 3.5 to 4.6 infections per 1000 workers, which is 2–4 times the level in the general population [6]. Worldwide, 66000 healthcare workers are infected by HBV each year through sharp injuries; with 261 deaths [7]. Prevention of HBV infection among healthcare workers is therefore a considerable challenge in health occupational safety. Moreover, prevention of HBV infection in healthcare workers is important to prevent its transmission to the patients they treat [8,9].

Since 1981 a vaccine against HBV is available. Considered safe and effective, this vaccine has a protective efficiency of 90–95% [10]. All health care workers are required to be vaccinated against HBV [10,11], especially theatre personnel such as surgeons and surgical trainees who are at a high risk of infection [12]. However the World Health Organization (WHO) has estimated that HBV vaccination coverage amongst healthcare workers is only 18-39% in low and middle-income countries compared to 67-79% in high-income countries [7]. To the best of our knowledge, in Cameroon there is no published information about HBV vaccination coverage among healthcare workers; among whom surgical residents constitute a special group, as they have been reported to have the highest occupational risk of HBV infection during health professional training [7].

This study sought to determine their knowledge of risk factors for HBV infection, awareness of HBV vaccine and the vaccination status of surgical residents in Cameroon. By surgical residents, we refer to medical doctors undertaking postgraduate training to become specialist consultants in any of the surgical specialties.

## Methods

This study was performed in accordance with the guidelines of the Helsinki Declaration and was approved by the Institutional Ethical Review Board of the Faculty of Medicine and Biomedical Sciences of Yaoundé. Written informed consent was obtained from all the participants.

This observational cross-sectional study was carried out in March 2012 at the Faculty of Medicine and Biomedical Sciences of the University of Yaoundé I, the only medical school in Cameroon that currently offers postgraduate medical training. Participants were recruited through a non-randomized, simple and consecutive sampling of eligible surgical residents met either at the campus of the faculty or the teaching hospitals during the period of the study. Data were collected using a structured pretested questionnaire whose validity and reliability were confirmed by Cronbach’s alpha coefficient (alpha = 0.74). The requested information was: socio-demographic and academic characteristics, knowledge of risk factors for HBV infection and HBV vaccine, history of accidental exposure to blood, HBV vaccination status, and perception of HBV vaccine.

We considered participants to be adequately vaccinated if they had received a minimum of three intramuscular injections of 20 micrograms of HBsAg (hepatitis B surface antigen) at a schedule of 0, 1 and 6 months; thus completing the minimum primary HBV vaccination series. Participants were considered inadequately vaccinated if they had started HBV vaccination but did not complete the three doses of primary vaccination, and not vaccinated if they had never received a dose of an HBV vaccine.

Data were coded, entered and analyzed using Statistical Package for Social Science (SPSS) version 20.0. The chi-square test or the Fisher’s exact test were used to compare qualitative variables, and a p value less than 0.05 was considered statistically significant.

## Results

There were 70 surgical residents at the medical school during the 2011–2012 academic year, 49 of whom are included in the present study. Among the 21 potential participants who were not included, 9 refused to participate because of lack of time, and 12 were not found during the study period. As shown in Table 1, respondents were predominantly male, in the first or second year of training, and Ear-Nose and Throat (ENT) or Obstetrics and Gynecology residents. Since the beginning of their residency program, 28 (57.1%) had had at least one accidental exposure to blood, with a median of 2 (range 1 to 25) exposures. There were no statistically significant differences observed in the prevalence of accidental exposure to blood between specialties (p = 0.212) and years of training (p = 0.355). Among the 28 residents who had had at least one accidental exposure to blood, only 15(53.6%) always notified the exposures and 11(39.3%) never considered the risk of HBV infection after exposure. However, all the 28 were aware of the risk of HIV infection from blood exposure and took post-exposure preventive measures to reduce the risk of HIV infection if the index patients were confirmed HIV-positive.

**Table 1:** Demographic and academic characteristics of the study population

**Characteristics**	**Number (%)**
Age (years)	
25-29	20 (40.8)
30-34	22 (44.9)
35-39	6 (12.2)
40-44	1 (2.0)
Sex	
Male	32 (65.3)
Female	17 (34.7)
Specialties	
General surgery	9 (18.4)
Pediatric surgery	1 (2.0)
Cardiac surgery	2 (4.1)
Orthopedics	4 (8.2)
Urology	3 (6.1)
Ob-Gyn	15 (30.6)
ENT	15 (30.6)
Academic level	
Year 1	16 (32.7)
Year 2	16 32.7)
Year 3	7 (14.3)
Year 4	10 (20.4)

Ob-Gyn, Obstetrics and Gynecology; ENT, Ear, Nose and Throat.

The majority of residents had good knowledge of risk factors for HBV infection (Table 2). Thirty-one (63.3%) recognized HBV as the most infectious blood-borne pathogen. Forty-eight respondents (98%) were aware of the existence of the HBV vaccine; 34 (69.4%) knew that a complete course of primary HBV vaccination includes three doses; and 33 (73.5%) were aware that an immune response test has to be conducted after the third dose of the HBV vaccine to verify if there was an adequate immunological response to vaccination.

**Table 2:** Knowledge of risk factors and HBV vaccination in the study population

Which is the most contagious blood-borne pathogen through AEB?	
HIV	9 (18.4)
HBV^a^	31 (63.3)
HCV	2 (4.1)
Do not know	7 (14.3)
Contact of healthy skin with infected blood is a risk factor of HBV infection	
Yes	11 (22.4)
No^a^	38 (77.6)
Do not know	0 (0)
Contact of abraded skin with infected body fluid is a risk factor of HBV infection	
Yes^a^	46 (93.9)
No	3 (6.1)
Do not know	0 (0)
Injury with needle contaminated with infected blood is a risk factor of HBV infection	
Yes^a^	48 (98)
No	1 (2)
Do not know	0 (0)
Contact of eyes with infected blood is a risk factor of HBV infection	
Yes^a^	47 (95.9)
No	2 (4.1)
Do not know	0 (0)
Can an infected surgeon contaminate its patient during the surgical intervention?	
Yes^a^	43 (87.8%)
No	5 (10.2%)
Do not know	1 (2.0%)
Does a vaccine against HBV exist?	
Yes^a^	48 (98)
No	1 (2.0)
Do not know	0 (0)
Minimum number of doses for a complete primary HBV vaccination	
1 dose	4 (8.2)
2 doses	6 (12.2)
3 doses^a^	34 (69.4)
4 doses	4 (8.2)
Do not know	1 (2.0)
Does an immune response test after HBV vaccination exist?	
Yes^a^	36 (73.5)
No	12 (24.6)
Do not know	1 (2.0)

Values presented are absolute counts (percentages); AEB, accidental exposure to blood; HBV, hepatitis B virus; HCV, hepatitis C virus; HIV, human immunodeficiency virus.

^a^Correct answer.

Although the majority of residents (89.8%) knew that they were more at risk of HBV infection than the general population (Table 3), and they were aware of the existence of the HBV vaccine (98%) and would recommend it to another resident (95.9%), only 12 (24.5%) were adequately vaccinated. In addition only 4 of the 12 (33.3%) adequately vaccinated residents had post-vaccination testing for antibodies against hepatitis B surface antigen, to confirm a good immunological response and thus effective protection against HBV infection. Eleven (22.4%) residents were inadequately vaccinated and 26 (53.1%) not vaccinated. Among the 26 (53.1%) residents who had never had any dose of the HBV vaccine, the main reasons for not being vaccinated were lack of time (38.5%), lack of money to pay for the vaccine (23.1%), and lack of sufficient information on the vaccine (19.2%). Only 10 (20.4%) residents had been informed by their training institutions of the importance of HBV vaccination. The residents who had been informed of the risk of HBV infection were not significantly more likely to be vaccinated than those who had not been informed (p = 0.73). Besides, as shown in Table 4, there was no significant statistical association between HBV vaccination status and age (p = 0.72), gender (p = 0.56), surgical specialties (p = 0.24), or years of surgical training (p = 0.13).

**Table 3:** Attitude towards HBV vaccine in the study population

Is HBV vaccine completely safe?	
Yes	21 (42.9)
No	24 (49)
Do not know	4 (8.2)
Are you more at risk of HBV infection than the general population?	
Yes	44 (89.8)
No	2 (4.1)
Do not know	3 (6.1)
Would you recommend HBV vaccine to another resident?	
Yes	47 (95.9)
No	2 (4.1)
Do you think that HBV vaccination should be compulsory for all HCWs at risk?	
Yes	45 (91.8)
No	4 (4.2)

Values presented are absolute counts (percentages); HBV, hepatitis B virus; HCWs, healthcare workers.

**Table 4:** HBV vaccination status according to the demographic and academic characteristics of the study population

**Characteristics**	**Not vaccinated or inadequately vaccinated**	**Adequately vaccinated**	**p value**
Age (mean (SD))	30.9 (2.8)	31.3 (4.8)	0.72
Sex (% M)			
Male	25	7	0.56
Female	12	5	
Specialties			
General surgery	7	2	0.24
Pediatric surgery	2	0	
Cardiac surgery	2	0	
Orthopedics	1	3	
Urology	1	1	
Ob-Gyn	12	3	
ENT	12	3	
Academic level			
Year 1	9	7	0.13
Year 2	14	2	
Year 3	5	2	
Year 4	9	1	

SD, standard deviation; M, men; Ob-Gyn, Obstetrics and Gynecology; ENT, Ear, Nose and Throat.

## Discussion

Surgeons and surgical trainees are at high risk of accidental exposure to blood and, consequently, HBV infection. HBV is very contagious, with a 30% risk of contamination after exposure by a single needle stick injury [2]. This risk is particularly high in countries like Cameroon with a high prevalence of HBV infection in the general population. In Cameroon, some studies have reported that HBV infection prevalence among blood donors, considered as apparently healthy adults, is around 15% [13,14]. Studies have reported highest occupational risk of HBV infection during health professional training [7]. In our study, approximately three-fifths of the respondents have had at least one accidental exposure to blood since the beginning of their training, with a median of 2 (range 1 to 25) exposures. Not reporting accidental exposure to blood increases the risk of HBV infection, since no post-exposure preventive measures are taken to reduce the risk of infection. In a study conducted by Robin Smith et al. in a London teaching hospital, 81% of the surgeons who were interviewed admitted that they did not always report accidental exposure to blood [15]. In our setting, 53.6% of the participants reported not notifying each accidental exposure to blood. Moreover, 39.3% of them had never considered the risk of HBV infection after exposure. All these data show how exposed surgical residents in Cameroon are to HBV infection.

Awareness of risk among healthcare workers is an important factor affecting HBV vaccine uptake [16-18]. Our study reveals that the majority of our respondents had good knowledge of the risk factors for HBV infection and almost all knew that they were more at risk of HBV infection than the general population. We also found that almost all of them were aware of the existence of HBV vaccine and would recommend it to another resident, but only one-quarter of them were adequately vaccinated. Moreover, we may have overestimated this proportion of the “adequately” vaccinated residents, because only 33.3% of the “adequately vaccinated” had an immune response test to confirm a good response of the HBV vaccine and thus an effective protection against HBV infection.

Comparable studies have consistently shown poor vaccination status among health care workers in low and middle-income countries [17,19-21]. Similar to our study, Kesieme et al. reported that only 26.8% of Nigerian theatre personnel were adequately vaccinated in spite of good knowledge of risk factors for HBV infection and of HBV vaccine, and willingness to recommend HBV vaccination to other healthcare workers, [21]. Several reasons may explain this low HBV vaccine uptake. Consistent with findings of Kesieme et al. [21], in our study the main reasons for not being vaccinated were lack of time to attend vaccination, lack of money to pay for the vaccine, and lack of sufficient information on the vaccine. Studies conducted in health institutions in which HBV vaccine is offered to healthcare workers have shown a significantly higher vaccine uptake [22,23]. However, as revealed elsewhere, making the vaccine accessible and free of charge may not be enough [24]. Other investigators have found refusal of HBV vaccine to be associated with the fear of side effects, including fear of getting HBV infection from the vaccination [16]. Half of the residents in our study expressed concerns about the safety of HBV vaccine, which could partially explain the low reported vaccination coverage.

In Chaudhari’s study in India, female healthcare workers were significantly more likely to be vaccinated than their male colleagues [24]. We did not find gender to be significantly associated with HBV vaccination status in our study. Moreover, age, academic level, and the specialty seemed not to influence HBV vaccine uptake in our study population. However, it is likely that our study was not adequately powered to detect significant differences with these factors. We also found that only one-fifth of the respondents had been sensitized by their training institutions on the importance of HBV vaccination. It should be compulsory for medical training institutions to recommend and make HBV vaccination accessible to their trainees; thus giving the trainees the opportunity to freely make an informed decision whether or not to take it.

## Conclusion

There is a low HBV vaccination coverage among surgical residents in Cameroon leading to continued occupational risk of HBV infection. To protect these trainees like other healthcare workers and their patients, complete HBV vaccination should be strongly recommended and offered to them before the beginning of their residency training.

## Competing interests

The authors declare that they have no competing interests.

## Authors’ contributions

JJNN designed the study, analyzed the data, drafted and revised the manuscript. JRNN designed the study design, collected and analyzed the data, critically reviewed and revised the manuscript. KKK collected the data, critically reviewed and revised the manuscript. AW and CSW critically reviewed and revised the manuscript. All the authors approved the final version of the manuscript.

## References

[B1] Calver J Occupational health services Am J Infect Control 1997 25 363 365 10.1016/S0196-6553(97)90078-X 9343616

[B2] Sarrazin U Brodt R Sarrazin C Zeuzem S Postexposure prevention after occupational exposure to HBV, HCV and HIV Unfallchirurg 2004 107 129 142 15049336 10.1007/s00113-004-0728-8

[B3] Barker LF Shulman NR Murray R Hirschman RJ Ratner F Diefenbach WC Geller HM Transmission of serum hepatitis. 1970 JAMA 1996 276 10 841 844 10.1001/jama.1996.03540100085042 8769597

[B4] Lavanchy D Hepatitis B virus epidemiology, disease burden, treatment, and current and emerging prevention and control measures J viral Hepat 2004 11 2 97 107 10.1046/j.1365-2893.2003.00487.x 14996343

[B5] Noubiap JJ Joko WY Nansseu JR Tene UG Siaka C Sero-epidemiology of human immunodeficiency virus, hepatitis B and C viruses and syphilis infections among first-time blood donors in Edéa, Cameroon Int J Infect Dis 2013 17 e832 e837 10.1016/j.ijid.2012.12.007 23317526

[B6] West DJ The risk of hepatitis B infection among health professionals in the United States: a review Am J Med Sci 1984 287 2 26 33 10.1097/00000441-198403000-00006 6369984

[B7] Prüss-Üstün A Rapiti E Hutin Y Estimation of the global burden of disease attributable to contaminated sharps injuries among health-care workers Am J Ind Med 2005 48 6 482 490 10.1002/ajim.20230 16299710

[B8] Gunson RN Shouval D Roggendorf M Zaaijer H Nicholas H Holzmann H de Schryver A Reynders D Connell J Gerlich WH Marinho RT Tsantoulas D Rigopoulou E Rosenheim M Valla D Puro V Struwe J Tedder R Aitken C Alter M Schalm SW Carman WF European Consensus Group Hepatitis B virus (HBV) and hepatitis C virus (HCV) infections in health care workers (HCWs): guidelines for prevention of transmission of HBV and HCV from HCW to patients J Clin Virol 2003 27 213 230 10.1016/S1386-6532(03)00087-8 12878084

[B9] Buster EH van der Eijk AA Schalm SW Doctor to patient transmission of Hepatitis B virus: implications of HBV DNA levels and potential new solutions Antiviral Res 2003 60 79 85 10.1016/j.antiviral.2003.08.014 14638402

[B10] Mahoney FJ Update on diagnosis, management and prevention of hepatitis B virus infection Clin Microbiol Rev 1999 12 2 351 366 10194463 10.1128/cmr.12.2.351 PMC88921

[B11] U.S. Public Health Service Updated U.S. Public health service guidelines for the management of occupational exposures to HBV, HCV, and HIV and recommendations for postexposure prophylaxis MMWR Recomm Rep 2001 50 RR-11 1 52 11442229

[B12] Taylor DL Bloodborne pathogen exposure in the OR--what research has taught us and where we need to go AORN J 2006 83 834 838 841–6; quiz 849–52 16674027 10.1016/s0001-2092(06)60004-5

[B13] Mbanya D Binam F Kaptue L Transfusion outcome in a resource-limited setting of Cameroon: a five-year evaluation Int J Infect Dis 2001 5 2 70 73 10.1016/S1201-9712(01)90028-1 11468100

[B14] Mbanya DN Takam D Ndumbe PM Serological findings amongst first-time blood donors in Yaounde, Cameroon: is safe donation a reality or a myth? Transfus Med 2003 13 5 267 273 10.1046/j.1365-3148.2003.00453.x 14617337

[B15] Smith ER Banatvala JE Tilzey AJ Hepatitis B vaccine uptake among surgeons at a London teaching hospital: how well are we doing? Ann R Coll Surg Engl 1996 78 447 449 8881729 PMC2502932

[B16] Doebbeling BN Ferguson KJ Kohout FJ Predictors of hepatitis B vaccine acceptance in health care workers Med Care 1996 34 1 58 72 10.1097/00005650-199601000-00005 8551812

[B17] Taalat M Kandell A El-Shoubary W Bodenschatz C Khairy I Oun S Mahoney FJ Occupational exposure to needle stick injuries and hepatitis B vaccination coverage among health care workers in Egypt Am J Infect Control 2003 31 8 469 474 10.1016/j.ajic.2003.03.003 14647109

[B18] Shim BM Yoo HM Lee AS Park SK Seroprevalence of hepatitis B virus among health care workers in Korea J Korean Med Sci 2006 21 1 58 62 10.3346/jkms.2006.21.1.58 16479066 PMC2733980

[B19] Adebamowo CA Odukogbe AA Ajuwon AJ Knowledge, attitude, and practices related to hepatitis B virus infection among Nigerian obstetricians and midwives J Obstet Gynaecol 1998 18 528 532 10.1080/01443619866255 15512169

[B20] Sofola OO Folayan MO Denloye OO Okeigbemen SA Occupational exposure to bloodborne pathogens and management of exposure incidents in Nigerian dental schools J Dent Educ 2007 71 6 832 837 17554101

[B21] Kesieme EB Uwakwe K Irekpita E Dongo A Bwala KJ Alegbeleye BJ Knowledge of hepatitis B vaccine among operating room personnel in Nigeria and their vaccination status Hepat Res Treat 2011 2011 157089 22028961 10.1155/2011/157089 PMC3199087

[B22] Vranckx R Jacques P De Schrijver A Moens G Hepatitis B vaccination coverage in Belgium health care workers Infection 2004 32 278 281 10.1007/s15010-004-2204-3 15624891

[B23] Ali NS Jamal K Qureshi R Hepatitis B vaccination status and identification of risk factors for hepatitis B in health care workers J Coll Physicians Surg Pak 2005 15 257 260 15907232

[B24] Chaudhari CN Bhagat MR Ashturkar A Misra RN Hepatitis B immunisation in health care workers MJAFI 2009 65 13 17 10.1016/S0377-1237(09)80046-4 PMC4921450 27408182

